# Application Research on Optimization Algorithm of sEMG Gesture Recognition Based on Light CNN+LSTM Model

**DOI:** 10.34133/2021/9794610

**Published:** 2021-11-08

**Authors:** Dianchun Bai, Tie Liu, Xinghua Han, Hongyu Yi

**Affiliations:** ^1^School of Electrical Engineering, Shenyang University of Technology, Shenyang 110870, China; ^2^Department of Mechanical Engineering and Intelligent Systems, University of Electro-Communications, Tokyo 182-8585, Japan

## Abstract

The deep learning gesture recognition based on surface electromyography plays an increasingly important role in human-computer interaction. In order to ensure the high accuracy of deep learning in multistate muscle action recognition and ensure that the training model can be applied in the embedded chip with small storage space, this paper presents a feature model construction and optimization method based on multichannel sEMG amplification unit. The feature model is established by using multidimensional sequential sEMG images by combining convolutional neural network and long-term memory network to solve the problem of multistate sEMG signal recognition. The experimental results show that under the same network structure, the sEMG signal with fast Fourier transform and root mean square as feature data processing has a good recognition rate, and the recognition accuracy of complex gestures is 91.40%, with the size of 1 MB. The model can still control the artificial hand accurately when the model is small and the precision is high.

## 1. Introduction

In recent years, surface electromyography (sEMG) has received great attention in driving prosthetic hand [1]. In order to realize the motor control using sEMG accurately, gesture action classification based on machine learning (ML) method, namely, pattern recognition (PR) and regression method based on classifier, has been widely studied. The regression model is mainly used for continuous wrist motion estimation [2], which can be used for synchronous control of multidegree of freedom (DOF), while the PR-based method uses discrete and sequential methods to distinguish gesture actions. Some ML-based regression methods, including linear regression (LR), random forest (RF), support vector regression (SVR), and artificial neural network (ANN), have been widely used in offline simulation and real-time control [3–7]. However, ML technology requires highly dependent feature extraction [8]. The appearance of convolutional neural network (CNN) provides a new method for feature learning and extraction through layer by layer processing [9, 10].

Jiang et al. propose a new method to extract specific knowledge from the data of motion time series by singular value decomposition (SVD) [11]. Bai and others proposed a recognition optimization method based on the combination of maximum mutual information channel selection, wavelet packet feature extraction, and support vector machine (SVM) and obtained more than 92% accuracy [12, 13]. Chen et al. proposed a new CNN structure composed of four convolution layers and one maximum pool layer. The structure is compact, the complexity of the model is reduced, and the accuracy of sEMG signal classification is improved [14]. Wei et al. proposed a multistream convolution neural network framework to improve the accuracy of gesture recognition by learning the correlation between a single muscle and a specific gesture [15].

Ding et al. proposed a parallel multiscale convolution architecture, which uses kernel filters of different sizes to recognize gesture actions [16]. Sun and others combine the generated flow model (GFM) with softmax classifier to classify gestures. The accuracy of classification of 53 different gestures in Ninapro database 5 is 63.86 ± 5.12% [17]. Although the spatial correlation of multichannel sEMG signals can be extracted by CNN, CNN ignores the time information in the continuous contraction of muscles. Recently, many researchers have begun to apply LSTM to the estimation of hand posture based on sEMG. Zhang et al. use LSTM to classify multimode gesture data collected by inertial measurement device, Myo armband (Thalmic Labs Inc.) and pressure sensor, which has achieved good results [18]. Teban et al. show that LSTM performs better than nonrecursive neural network in the nonlinear mechanism control of human hand [19]. In He et al.'s study, combining LSTM with ANN, sEMG dynamic and static information is used to identify sEMG signals [20]. Although many scholars use convolutional neural network and long-term memory network to achieve better results in the relationship between muscles and gesture movement classification of sEMG signals, the size of the model is not considered. Because of the cost problem in the current stage, the flash in microprocessor is limited, and most of the deep learning training models are difficult to be applied in embedded hardware.

In order to solve the problem that the deep learning model generated in the process of gesture recognition, using sEMG signal is too large to be applied. Inspired by the advantages and limitations of CNN and LSTM, this paper proposes a CNN+LSTM hybrid framework. The CNN+LSTM hybrid model effectively combines the deep feature extraction of machine learning with sequence regression and makes full use of the spatiotemporal correlation of sEMG, so as to generate a smaller deep learning model with high accuracy. By using the deep features extracted by CNN and LSTM unit operation, complex gesture signals can be predicted accurately. Compared with traditional CNN, CNN+LSTM has stronger robustness to local distortion, and the generated prediction model is smaller and accurate. In this study, ten healthy participants participated in a series of wrist movement experiments. Experimental results show that the performance of CNN+LSTM is superior to CNN and traditional machine learning methods. When complex hand motion is activated in multidegree of freedom, this advantage will be more obvious.

The rest of this paper is structured as follows.  Section 2 introduces the experimental equipment and data acquisition.  Section 3 describes the data processing and the proposed CNN+LSTM hybrid framework and how to lighten and quantify the model. In Section 4, after optimizing the training model, the training results of the deep learning training model are given. The fifth part is the conclusion and puts forward the future work.

## 2. Materials and Methods

**Figure 1:**
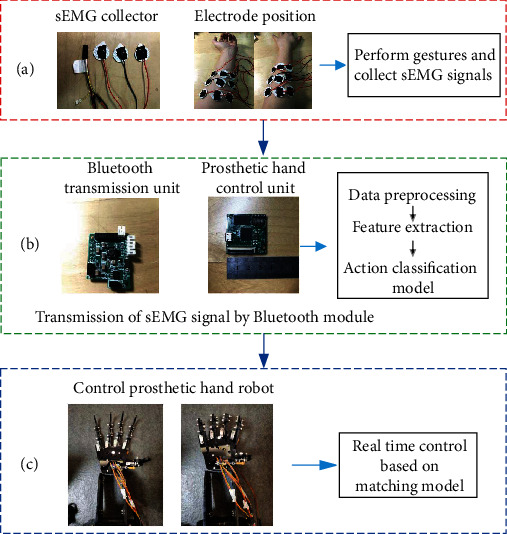
sEMG collector and prosthetic hand control flow chart.

The data acquisition device in this experiment is a self-developed sEMG acquisition module, which can accurately collect human upper limb sEMG signals, the sampling frequency is 2 K Hz, and the actual physical map is shown in Figure 1(a); the experimental data source is the 10 healthy subjects including 4 females and 6 males, aged between 23 and 26 years old, with an average age of 24.3 (±1.03) years, a height of 172.1 (±6.46) cm, and a weight of 71.3 (±9.58) kg. The subject had not received training before the test. At the beginning of the experiment, each subject sat on a comfortable chair and relaxed his arms. The collected sEMG signals are transmitted to the hardware experimental platform through Bluetooth for data preprocessing and model matching, and the hardware experimental platform can control the steering gear. The actual physical diagram is shown in Figure 1(b).

The prosthetic hand experimental platform is shown in Figure 1(c). As shown in Figure 1(c), the 5 fingers of the artificial hand experimental platform are controlled by 5 servos, but they do not have the freedom of the wrist joints. In the later stage, the deep learning model needs to be imported into the hardware experimental platform for real-time control. This hardware experiment platform is based on the microprocessing chip STM32. Its internal flash is 2 M, RAM is 16 M, and the main frequency is up to 480 MHz. When SRAM is off and in standby mode, the power consumption of the chip is 2.95 *μ*A. When VDD = 3.3 V and the temperature is 25°C, the power consumption of the chip is 263 *μ*/MHz, with good performance, and the microprocessor has an excellent price/performance ratio.

**Figure 2:**
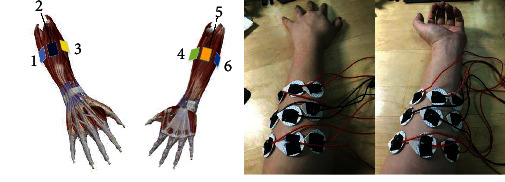
Schematic diagram and physical map of EMG collection location.

Figure 2 shows the location of the electrode and the actual picture of the position of the electrode. As shown in Figure 2, 18 electrodes were placed at the proximal forearm to collect sEMG signals in 6 channels. Each of the three electrodes forms a path, two of which are differential signals and one of which is reference ground signal. In order to obtain better differential effect, the two differential electrodes are placed on both sides and the reference electrode is placed in the middle. In addition, in order to reduce crosstalk, the distance between the two differential electrodes is about 30 mm. The EMG signals of extensor digiti minimi, extensor digitorum, abductor digitorum longum, flexor digitorum longum, flexor digitorum superficialis, and flexor digitorum profundus were collected in sequence.

**Figure 3:**
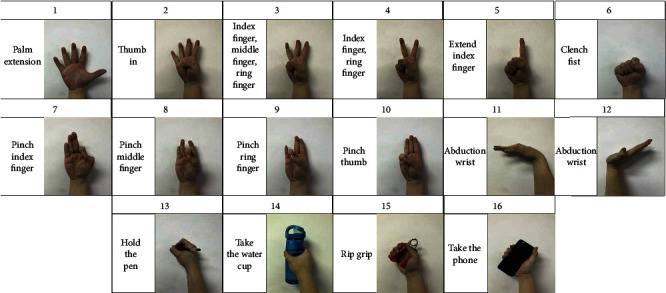
Illustration of sixteen gesture actions.

As shown in Figure 3, in the experiment, participants are required to implement 16 predefined gesture motion protocols. Each experimenter conducted an experiment in the order shown in Figure 2, with each action performed three times, and each action had one minute to rest. Since the measured sEMG signal is almost harmless to the subjects, participants can stop the experiment at any time to prevent any discomfort. The upper limbs should be placed horizontally on the table, the elbows should be slightly supported, the palms should be downward, and the fingers should be slightly bent. All operations start from this static position. Because the data will be scrambled before deep learning, the sequence of 16 kinds of gestures will not affect the results.

The original sEMG signal is collected by the sEMG signal acquisition chip in Figure 1(a), and the collected original sEMG signal will be stored in csv format. The training platform of the experimental depth model is the Dell workstation host, equipped with 2080Ti graphics card. The original sEMG signal needs to be preprocessed before deep learning, The processed data is generated into a small deep learning model through the light CNN+LSTM hybrid structure and imported into the left platform of Figure 1(b). On this experimental platform, data processing and feature extraction are carried out, and the gesture signals are classified, so as to facilitate the false hand control of the right platform of Figure 1(b).

## 3. Data Processing and Lightweight CNN+LSTM Hybrid Structure

In the existing deep learning data processing stage, time-domain analysis is simple and easy to perform in the deep learning data processing stage, but the disadvantages are long training time, high hardware requirements, poor real-time performance, and only time-domain analysis ignores frequency-domain analysis, It will cause a lot of eigenvalues to be lost, and it is impossible to perform deep learning accurately to achieve model matching. This research mainly combines the time-frequency-domain analysis with the time-domain analysis of the surface EMG signal. After the original sEMG signal is processed in the time-frequency domain, the amplitude after the time-frequency-domain transformation is processed to generate the eigenvalue matrix. The CNN network extracts feature vectors and trains the extracted feature vectors on the LSTM model to generate a gesture prediction model. This combination of time and frequency domains and the use of the CNN+LSTM hybrid model to generate a model are smaller and more accurate. After the original sEMG signal is processed as described above, its characteristic value will be easy to analyze, so as to generate a predictive model more accurately, which is more conducive to the control of the prosthetic hand in the later stage.

### 3.1. Feature Extraction

The experimental model uses sliding window technology for feature extraction, the data set is divided into data segments, and the sliding window with a length of 100 is used to divide the data. In order to improve the real-time accuracy, the step size of two consecutive sliding windows is set to one point (5 ms). In the process of feature extraction in the sliding window, in order to reduce the amount of computation, only the combination of root mean square (RMS) and fast Fourier transform (FFT) is selected in time-domain processing. The time-domain features selected in this experiment are root mean square (RMS), mean acceleration (MAV), waveform length (WL), zero cross (ZC), and slope sign change (SSC). In order to facilitate the study, the time window is 100, the step size is 100 (no overlap), and the number of sampling points is 100 in the FFT of time-frequency domain.

- MAV

MAV is one of the most commonly used parameters in sEMG signal analysis. The feature of MAV is the average of absolute amplitude of sEMG signal in sliding window. It provides information about the level of muscle contraction. *s*(*k*) is the *k*th amplitude sample, and *N* is the sample size. MAV can be calculated as *R*.

(1) $$\begin{array}{c} R=\sqrt{\frac{1}{N}\overset{N}{\underset{k=1}{\sum }}s{\left(k\right)}^{2}}. \end{array}$$

(b) WL

WL is another method to represent the frequency information of surface EMG signal, which represents the waveform characteristics of sEMG signal graph.

(2) $$\begin{array}{c} WL\left(x_{i}\right)=\overset{L}{\underset{k=1}{\sum }}\left|x_{i,k}-x_{i,k-1}\right|. \end{array}$$

(c) SSC

SSC is one of the commonly used parameters in sEMG signal analysis. It is defined as *s* in the equation and represents the number of times the slope symbol changes in the sliding window.

(3) $$\begin{array}{c} S=\overset{N-1}{\underset{k=2}{\sum }}\left|\left(s\left(k\right)-s\left(k-1\right)\right)\times \left(s\left(k\right)-s\left(k+1\right)\right)\right|. \end{array}$$

(d) ZC

ZC is the number of zero crossing. This feature calculates the frequency of surface EMG signal passing through the zero point. At the same time, it is set to reduce the influence of noise in the signal.

(4) $$\begin{array}{c} ZC=\left(\left|x_{i,k}-x_{i,k-1}\right|\geq \varepsilon \right). \end{array}$$

(e) RMS

RMS represents the average power of the surface EMG signal, reflecting muscle activity. It is defined as *R* in formula (5):

(5) $$\begin{array}{c} R=\sqrt{\frac{1}{N}\overset{N}{\underset{k=1}{\sum }}s{\left(k\right)}^{2}}. \end{array}$$

In this study, the original EMG signal is preprocessed before deep learning. The original data of EMG signal is processed by MAV, WL, SSC, ZC, RMS, FFT, and FFT + RMS, respectively. After processing, the original data will generate multidimensional characteristic data matrix as the input of deep learning model. Due to the huge amount of data, the generated multidimensional characteristic data matrix is stored in h5 file format, and the file generated by h5 file with its special data structure has small space. It can be easily imported into the deep learning model, and the transmission between each deep learning layer is in the form of matrix. In order to improve the accuracy of classification, in addition to using the above feature parameters, this experiment extracts the preprocessed signal through sliding window.

### 3.2. Preprocessing of Data

In the experiment, a third-order Butterworth high-pass filter (20 Hz) was used to eliminate motion artifacts [21], and a low-pass filter (2 KHz) was used to remove high-frequency noise. At the same time, a 50 Hz notch filter is used to reduce power line noise. Apply min-max scaling to normalize the surface EMG signal in each channel [22].

Because the sEMG signal is a nonstationary signal, the pretreatment method of sEMG is usually time-domain analysis. In this paper, the method of combining time-frequency and time domain is adopted in the data preprocessing stage, the fast Fourier transform is used to make the sEMG fast Fourier transform first, and then, the sEMG signal is processed twice by the way of time-domain processing. Fourier transform allows frequency-based signal analysis. However, in essence, when the signal is a nonstationary signal, Fourier transform cannot work normally. Therefore, it is limited to analyze these signals by Fourier transform. One of the technologies to solve this problem is fast Fourier transform (FFT), which is called the efficient and fast calculation method of computing discrete Fourier transform (DFT) by computer. The basic idea of FFT is to decompose the original *N*-point sequence into a series of short sequences in turn. The symmetry and periodic properties of the exponential factors in DFT are fully utilized, and then, the corresponding DFT of these short sequences is calculated and combined appropriately to delete the repeated calculation, reduce the multiplication operation, and simplify the structure. The formula of DFT is

(6) $$\begin{array}{c} f\left(x\right)=a_{0}+\overset{\infty }{\underset{n=1}{\sum }}\left(a_{n}\cos\frac{n\pi x}{L}+b_{n}\sin\frac{n\pi x}{L}\right). \end{array}$$

**Figure 4:**
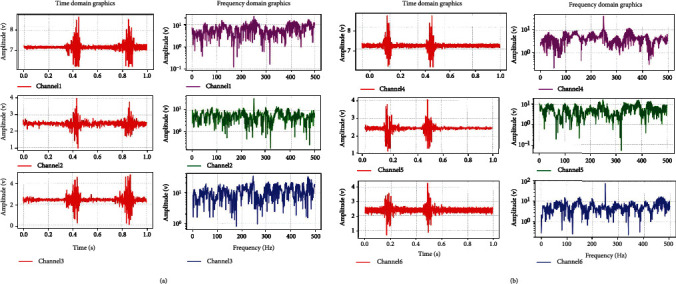
(a) sEMG of 1-3 pathway after palmar extension twice (left) and surface electromyography after fast Fourier transform (right). (b) sEMG of 4-6 pathway after palmar extension twice (left) and surface electromyography after fast Fourier transform (right).

In the original signal processing stage, in order to retain the characteristics of the original sEMG signal to the greatest extent, the fast Fourier transform is used to extract the time-frequency characteristics of the sEMG signal. The data collector of this experiment is 2 KHz. In the fast Fourier transform, 100 signal values are selected each time, the window length of FFT is 100, and the step length is 100. The results of some original signals and FFT are shown in Figure 4.

### 3.3. Construction and Training of 2D CNN+LSTM

**Figure 5:**
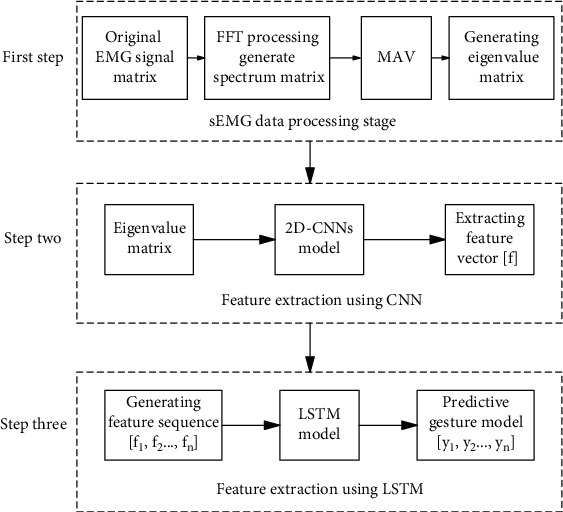
Block diagram of CNN+LSTM hybrid framework for data processing.

As shown in Figure 5, the 2D CNN+LSTM model includes two steps: the first step is to realize CNN feature extraction. First, CNN is used to extract deep feature vectors from multichannel sEMG signals. In the second step, LSTM unit is used to generate gesture prediction model. Firstly, the continuous deep feature vectors are arranged into a series of feature sequences, for example, [*f*_1_, *f*_2,_ · ⋯, *f*_*k*_], where *f*_1_ is the first feature map feature generated after the first convolution of convolution neural network after the first sampling, and *f*_2_ is the second feature map feature generated after the second convolution of convolution neural network after the second sampling, where *f*_*n*_ is the *n*th feature map feature generated after the *n*th convolution, denoted as [*f*] in Figure 5, and parameter *n* is the number of feature vectors in a feature sequence, which represents the time steps of recursive regression. LSTM is used to convert [*f*_1_, *f*_2,_ · ⋯, *f*_*n*_] into gesture action [*g*_1_, *g*_2_, ⋯*g*_*k*_], where *g*_1_ is gesture action 1 shown in Figure 3 and *g*_2_ is gesture action 2 shown in Figure 3. In this study, *k* = 16, and the final output is used as the final observation target of the sequence. The following will introduce in detail the CNN feature extraction and LSTM gesture prediction, as well as the training process of the hybrid model.

#### 3.3.1. Deep Feature Extraction Based on CNN

**Figure 6:**
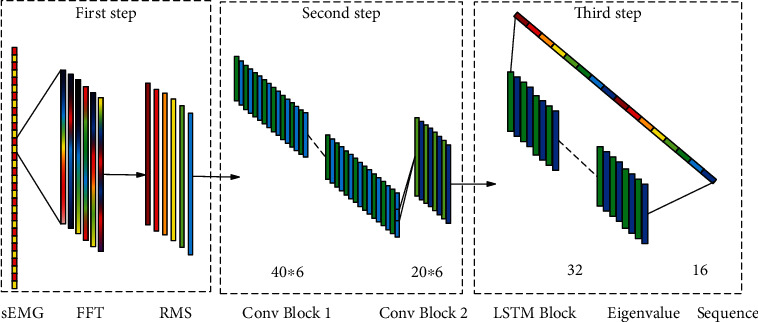
Structure diagram of the CNN+LSTM hybrid model.

*Construction of sEMG matrix*: as shown in the first step of Figure 6, firstly, the multichannel sEMG signal is divided into several segments by using the sliding window method with the window of 200, and then, a segment of the signal is arranged into 200 × six × 1 matrix. This corresponds to the length of the sliding window and the number of sensor channels. The amplitude of each spectrum can be obtained by fast Fourier transform (FFT) of each channel. The amplitude of each spectrum can be regarded as a time series. After time-domain processing of MAV, the sEMG matrix based on multifeature data can be obtained as the input of CNN.

*CNN architecture*: as shown in Figure 6, the CNN is composed of 2 convolution blocks (Conv blocks). The Conv block has a convolutional layer and a max pooling layer. The convolutional layer uses boundary padding with a kernel size of 4. There are 40 kernels in the first convolution block and 20 kernels in the second convolution block.

#### 3.3.2. LSTM-Based Sequential Regression

*Topology of LSTM*: LSTM is a network used to encode context information of time series with feedback loop. It contains the period of network activation input from the previous time point to influence the prediction of the current time point [23].

*LSTM architecture*: as shown in the third step of Figure 5. The LSTM used in this paper consists of one LSTM operation unit. The operation unit has a flatten layer for dimension reduction, a dropout layer, and a full connection layer, and the leaky relu layer is used to solve the dying relu problem [24].

#### 3.3.3. CNN+LSTM Training

In this study, the idea of training CNN+LSTM model at the same time is adopted. The main reason is that this strategy in training CNN and LSTM can save more computer space and improve operation efficiency. In addition, a component (CNN or LSTM) can be changed at the same time before training the model, which is more flexible in practical application. Specifically, CNN and LSTM are tuned in two steps. Firstly, a regression layer is added to the proposed CNN architecture to complete supervised learning. In this step, the input of the model is sEMG data. Secondly, deep feature vectors are extracted from the processed matrix of the second convolution block of CNN, and on this basis, feature sequences are constructed to train LSTM for sequence regression. The specific hybrid model architecture is shown in Figure 6.

*CNN network training setting*: the training method in this network is random momentum gradient descent method (SGDM). The verification frequency is set to twice per epoch. The maximum batch size is set to 128 because lower batch values increase training time. These layers and parameters are selected empirically.

*LSTM network training setup*: in this study, the duration of regression sequence was set to 1 second. In practice, the trade-off between the amount of time-dependent information and the amount of computation is realized. Adaptive moment estimation (ADAM) is used to train LSTM in 32 small batches in 200 periods. The dynamic learning rate was initialized to 0.01 and decreased by 80% after every 20 cycles. Therefore, only one LSTM layer and 50 hidden units are used in this study. For regularization, an exfoliation layer with 20% exfoliation rate is added.

Gesture classification based on surface electromyography includes intraexperiment and interexperiment evaluation. In order to realize the in experiment evaluation, the data in a test of each protocol is divided into three parts. The first two parts are used for model training, and the last part is used for testing. In order to avoid data leakage, it should be split before data preprocessing. In the interexperiment evaluation, one complete experiment is used for model training and the other is used for testing in the same protocol. This method can better verify the robustness of the model to the time-varying surface EMG signal. In this study, the deep learning model uses the crossentropy function as the loss function to ensure that the loss size is 0.01 in the training stage of the data set and 0.1 in the verification set.

### 3.4. Implementation and Size Evaluation of CNN+LSTM Lightweight Model

In the deep learning model training of this experiment, compared with the traditional deep learning process, the CNN training process is improved. Taking the deep learning model as 2D CNNs+LSTM as an example, after twice convolution neural network feature extraction, it is not directly connected with the full link layer but directly transforms the processing results into the dimension of the feature matrix by using reshape unit and takes the processed results as the input of LSTM. This method can effectively reduce the intermediate variables generated by the full link layer in the process of convolutional neural network, can quickly perform calculations, and make the trained model smaller, thereby saving hardware space.

The calculation of parameters of deep learning model can estimate the size of the model generated by deep learning; *N*^0^ represents the size of the input layer and is defined as

(7) $$\begin{array}{c} N^{0}=N_{0}*C_{0}*H_{0}*W_{0}. \end{array}$$

*N*_0_ represents the number of input images; *C*_0_ and *H*_0_ represent the length and width of convolution kernel, respectively; and *W*_0_ represents the number of output graphics. In the first convolution operation *N*_0_ = 1.

In order to estimate the size of the experimental model, we take the deep learning model 2D CNN+LSTM as an example. As shown in Figure 5, when the input is 200∗6∗1, the convolution core size in the first layer convolution neural network is 40∗6, and the output is set to 128 feature maps. The second convolution kernel is 20∗6, and the output is set to 8 feature maps. Because in LSTM training, it is necessary to convert the three-dimensional data into two-dimensional data, that is, to the size of 181∗8, select the size of LSTM as 32 for training. According to formula (7), the size of the first layer convolutional neural network is 30720. According to formula (7), the size of the second layer convolution neural network is 122880. The size after dimension transformation is 1448. The input size of LSTM network is 46336. The variables produced in the training process are 128, 20, and 32. The total parameter size of deep learning training is 201564, and the number of bytes is 787.36 kb. Compared with the actual training model, the size of the model is 909 kb, which is basically close, because there may be additional characteristic parameters in the model.

## 4. Discussion

This experiment mainly conducts research from three aspects, discussing the selection of traditional machine learning feature values, the size of the trained model, and the accuracy of complex gesture training. First, in order to solve the problem that the traditional deep learning training time is too long and the model is too large to be convenient for application, this paper conducts an experimental analysis on the selection of deep learning feature values, then uses the selected feature values to compare the accuracy and model of different deep learning models. The size of the research is carried out to obtain a smaller high-precision training model; finally, the deep learning model trained in the experiment is combined with the prosthetic hand control experimental platform to realize the real-time control of the prosthetic hand.

### 4.1. Select the Comparison between Different Eigenvalues

**Figure 7:**
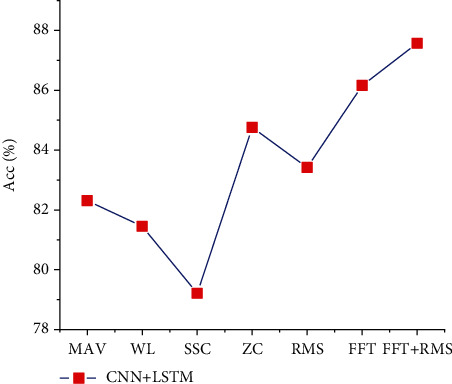
Training results of the CNN+LSTM training model with different eigenvalues.

The characteristic data selected in this experiment are the mean absolute value of sEMG amplitude (MAV), the cumulative length of sEMG waveform (WL), the number of changes of sEMG amplitude slope (SSC), the area center value of sEMG signal (ZC), the average power of sEMG signal (RMS), and the fast Fourier transform (FFT) of sEMG signal. The combination of fast Fourier transform (FFT) and root mean square (RMS) of amplitude (FFT+RMS) of surface EMG signal means that the real time-domain features are RMS, MAV, WL, ZC, and SSC; the time-frequency-domain feature data is FFT; and the time-frequency-domain feature data is combined with the time-domain feature data (FFT+RMS). In order to facilitate the study, the time window is 100, the step size is 100 (no overlap), and the number of sampling points is 100 in the FFT of time-frequency domain. In the selection of deep learning model, 1D CNN+1D LSTM is selected as the training model. In this training model, the convolution core of CNN is 40∗6, and the size of LSTM is 32.  Figure 7 shows the test set accuracy of different characteristic data under the same training model and the same acquisition window.

As shown in Figure 7, the training results of time-domain eigenvalues are lower than those of time-frequency eigenvalues, and the combination of time-frequency and time-domain eigenvalues is better as the training results of feature values. That is, the characteristic value of RMS is carried out after FFT transformation of the original sEMG signal. The training accuracy of the model is higher than that of the original surface EMG signal after time-domain transformation and only time-frequency transformation. The main reason is that the time-domain transformation only considers the characteristic value of sEMG signal in time domain, but ignores the frequency-domain characteristic; in the process of sEMG signal processing, the time-frequency-domain transform does not lose the frequency-domain characteristic of the signal, and the speed is faster and the effect is better. The amplitude is processed in time domain, and the amplitude characteristics of the surface EMG signal are fully utilized, which provides a good number for the further study model.

### 4.2. Comparison of Accuracy of Different Depth Learning Models

**Figure 8:**
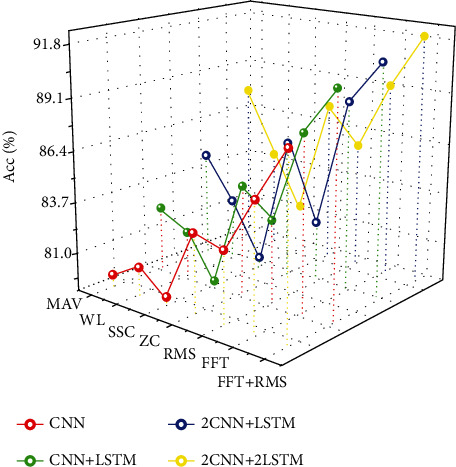
Training accuracy of each eigenvalue under different training models.

In this experiment, four deep learning models are selected for comparison: CNN, CNN+LSTM, 2D CNN+LSTM, and 2D CNN+2D LSTMs. The selected characteristic data are MAV, WL, SSC, ZC, RMS, FFT, and FFT+RMS. The training results of each model are shown in Figure 8. The red solid line indicates the accuracy of CNN input test set with different characteristic data, and the convolution kernel of CNN in this training model is 40∗6. The green solid line indicates the test set accuracy of CNN+LSTM inputting different characteristic data. In this training model, the convolution kernel of CNN is 40∗6, and the size of LSTM is 32. The blue solid line indicates the accuracy of the test set of 2D CNN+LSTM input different characteristic data. In this training model, the first layer convolution kernel of CNN is 40∗6, the second layer convolution kernel is 20∗6, and the LSTM is 32. The yellow solid line represents the test set accuracy of 2D CNN+2D LSTM with different input characteristic data. In this training model, the first layer convolution core of CNN is 40∗6, the second layer convolution core is 20∗6, the first layer LSTM is 32, and the second layer LSTM is 16.

As shown in Figure 8, when MAV, WL, SSC, ZC, RMS, FFT, and FFT+RMS are selected as eigenvalues, the training effect of time-frequency-domain eigenvalue FFT+RMS is better than that of time-domain eigenvalue and frequency-domain eigenvalue, and the deep learning training model of 2D CNNs+2D LSTMs is generally better than the other three training models. When FFT+RMS is used as the eigenvalue, the training accuracy of each model is higher than that of only using a single eigenvalue. Comparing the accuracy of the whole model training, using FFT+RMS as eigenvalues for deep learning, no matter what kind of deep learning model structure is used, it is higher than using only time-domain eigenvalues and frequency-domain eigenvalues.

Based on the above experimental results, we can draw a conclusion: in the selection of eigenvalues, the time-frequency-domain eigenvalues are more accurate than the time-domain eigenvalues, and the highest accuracy rate is obtained when the eigenvalues are combined with fast Fourier transform and root mean square; in the selection of deep learning model, the accuracy rate is not ideal when the model is only single-layer CNN; the subsequent experiment is carried out in the single-layer CNN model. In order to further improve the accuracy, we continue to add a layer of CNN model on the basis of CNN+LSTM, which is used to extract feature signals and train, and the experimental accuracy continues to increase, however, when the experiment is in the later stage of 2 CNNs+LSTM. After adding a layer of LSTM model on the basis of LSTM, the accuracy of the experiment has increased, but the improvement is not too large. The 2D CNN+2D LSTM model has the highest accuracy in different depth learning models.

### 4.3. Size Comparison of Different Eigenvalue Generation Models

**Figure 9:**
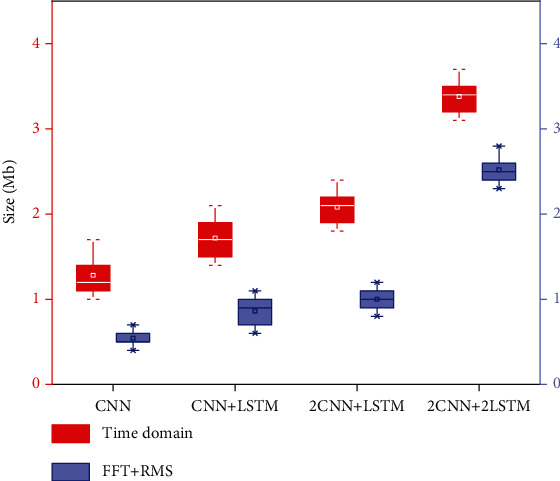
Size comparison of different eigenvalue generation models.

In this experiment, we need to import the training model generated by deep learning into the hardware platform, so as to model match the hand EMG signal and realize the control of the prosthetic hand. The flash of the prosthetic hand control hardware platform is 2m, which requires that the experimental model generated through deep learning should be less than 2m. In order to import the training model into the experimental platform, the size of the experimental model generated by different preprocessed feature data is compared, as shown in Figure 9, which is the comparison diagram of the size of the experimental models generated in different depth learning models by combining time-domain eigenvalues and time-domain eigenvalues (FFT+RMS). Red represents the size of the experimental model generated by each time-domain eigenvalue, and blue represents the size of the model generated by combining time-domain eigenvalues and time-domain eigenvalues in different sampling windows.

According to the comparison results in Figure 9, the training model generated by using time-domain feature data in different depth learning training structures is larger than that of time-frequency-domain and time-domain combination. Moreover, the training precision of the model trained by time-domain feature data when the deep learning structure is CNN is shown in Figure 8. When the feature data is MAV, WL, SSC, ZC, and RMS, the accuracy of test sets is 79.63%, 80.52%, 79.41%, 83.25%, and 82.79%; that is, when the depth learning model is CNN, the average value of selecting time-domain characteristic data is 81.12%, and when the model structure of the deep learning model is CNN+LSTM, 2CNN+LSTM, and 2CNN+2LSTM, the model size is greater than 1.5 M, which is not suitable for importing to the artificial hand control hardware platform, so according to the above analysis, The experimental model of time-domain feature data generation cannot be applied on this experimental platform.

**Figure 10:**
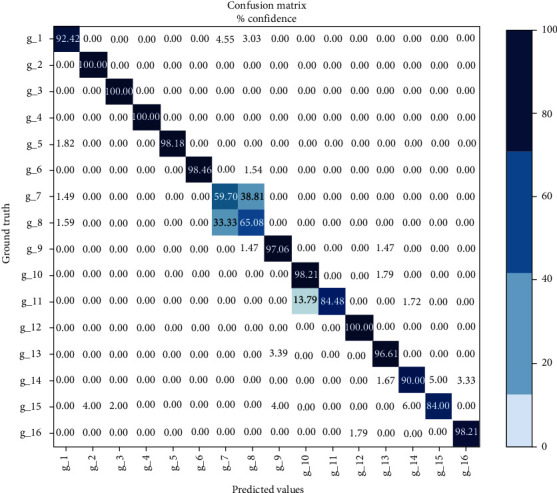
Confidence matrix of 2D CNN+LSTM gesture training results.

According to the comparison results in Figure 9, when using the combination of time-frequency domain and time-domain (FFT+RMS) as eigenvalues, the overall training model of deep learning is small. When the deep learning structure is CNN, the accuracy is 87.47%, and the model size is about 0.5 MB; when the deep learning structure is CNN+LSTM, the accuracy is 89.57%, and the model size is about 0.7 MB; but the accuracy of the above two deep learning models is low. Although it is high, the accuracy is not ideal for real-time control. When the structure of deep learning model is 2D CNN+LSTM, the accuracy is 91.25%, and the model size is about 1 MB. Compared with the first two deep learning models, the accuracy is higher, and the model is less than 2 MB. Although the accuracy of deep learning model structure is 2D CNN+2D LSTM and the model is more than 2 M, it cannot be applied on hardware platform. In conclusion, the deep learning model of the real-time prosthetic hand control experimental platform is 2D CNN+LSTM, and its model size is about 1 MB; the training accuracy is high.  Figure 10 shows the confidence matrix of 16 gesture training results with the deep learning model of 2D CNN+LSTM.

The confidence matrix in Figure 10 shows that the accuracy of the second, third, fourth, and twelfth gesture models is 100%, and the accuracy of the fifth, sixth, ninth, tenth, and thirteenth gesture models is about 98%. The accuracy rate of category 16 is over 96%, and that of category 1, 11, 14, and 15 is over 84%. Only the accuracy rate of categories 7 and 8 is about 60%. It can be seen from the above figure that the accuracy of this training method in pinching the index finger and middle finger needs to be improved, but for other types of gesture models, the accuracy is more than 84%.

For the problem of low accuracy of the seventh and eighth categories, as shown in the position pasted by the electrode sheet in Figure 1(a), the electrode sheet detects the sEMG signal of the extensor finger at the second position, and the extensor finger could cause the extension of the index finger and middle finger at the same time, which makes it difficult to distinguish the sEMG signals of the index finger and middle finger, resulting in low accuracy of the seventh and eighth categories.

### 4.4. Using Training Model to Control Prosthetic Hand

**Figure 11:**
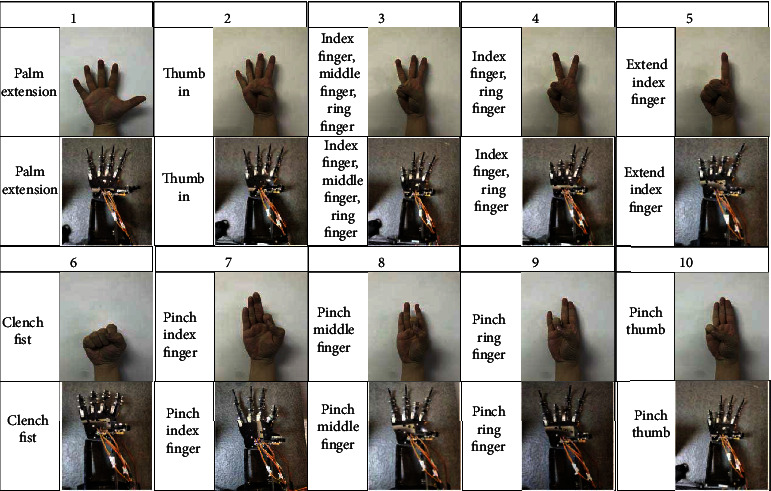
Schematic diagram of prosthetic hand with 10 gestures.

In this experiment, the model of 2D CNN+LSTM is used to control the artificial hand by introducing the model into the developed hardware experimental platform. The model can match the hardware experimental platform in size and accuracy. The artificial hand experiment platform can control the complex finger movements accurately. The movement pattern of each part of fingers is shown in Figure 11.

As shown in Figure 11, the depth training model trained by 2D CNN+LSTM network structure can analyze sEMG signals in offline mode and accurately control the finger joints of the prosthetic hand. Because the wrist movement is included in the training model, the prosthetic hand experimental platform does not have wrist freedom, and the prosthetic hand control can only carry out finger movement.

In this group of experiments, the results of different processing of sEMG signals are used as the characteristic data of deep learning, and the accuracy of the trained model is compared according to different characteristic data. Under the same deep learning model structure, the combination of fast Fourier transform and average power as the input of feature data is about 3.85% higher than other feature data, especially for sEMG data containing finger motion. This advantage comes from the strong ability of fast Fourier transform to obtain the time-domain and frequency-domain information of muscle activity from the original sEMG signal, Secondly, with the increase of deep learning network structure, the time required for learning and recognition will increase rapidly, and the hardware space occupied by the model also increases. Therefore, there is a compromise between recognition accuracy and model size. Due to the limited flash size on the hardware system platform in the current market, it is necessary to find a deep learning model with high accuracy and small model. When 2D CNNs+LSTM is used as the training model, the accuracy is 2.25% higher than other training models, and the generated training model is small, which can be applied on the developed hardware test platform. In the later stage of the experiment, the generated experimental model is used to control the prosthetic hand experimental platform, and good experimental results are obtained. Gesture recognition and control technology based on two-dimensional artificial neural network (CNN) and long-term and short-term memory network (LSTM) technology will be a promising control technology. In addition, compared with the deep neural network used in other studies, the 2D CNN+LSTM structure used in this study is relatively simple and easy to apply.

## 5. Conclusion and Prospect

In this paper, a light sEMG gesture recognition algorithm based on convolutional neural network and long-term and short-term memory network is proposed. We use self-made sensors to obtain surface EMG signals, apply different processing methods to extract features, and compare the training accuracy of different depth learning models. In the deep learning model training, a light CNN+LSTM training method is constructed. The training model has high accuracy, the size is about 1 MB, and it can control the prosthetic hand in embedded hardware. At the same time, the recognition accuracy of the model is 91.25%, which is higher than the existing recognition algorithms.

In the later experiments, we will consider increasing the number of channels to solve the problem of low recognition of the seventh and eighth types of gestures, continue to optimize the algorithm, strive to improve the accuracy of each gesture in deep learning, and keep the training model small, so that it can be better applied on the hardware platform.

## Data Availability

The sEMG data used to support the findings of this study are available from the corresponding author upon request.
